# Recurrence and mortality following treatment for Clostridum difficile infection with metronidazole or vancomycin

**DOI:** 10.1186/2047-2994-4-S1-O38

**Published:** 2015-06-16

**Authors:** V Stevens, R Nelson, K Khader, M Jones, K Brown, M Samore, M Rubin

**Affiliations:** 1 IDEAS 2.0 Center, Veterans Affairs Salt Lake City Health Care System (VASLCHCS), Salt Lake City, UT, USA

## Introduction

Metronidazole is considered first-line therapy for patients with mild to moderate *Clostridium difficile* infection (CDI), but was recently shown to be inferior to vancomycin for clinical cure. The effect of treatment choice on downstream outcomes such as recurrence and risk of death is not well understood.

## Objectives

To evaluate the impact of vancomycin or metronidazole on CDI recurrence and 30-day mortality.

## Methods

A retrospective cohort study of patients with CDI who were treated with vancomycin or metronidazole in the US Department of Veterans Affairs healthcare system between 1 January 2005 and 31 December 2012. A diagnosis of CDI was based on a positive enzyme immunoassay or cytotoxin test. CDI treatment was defined as administration or dispensing of vancomycin (oral or compounded) or metronidazole (oral or intravenous) within two days before or after the positive CDI result. Each patient treated with vancomycin was matched with up to four patients treated with metronidazole on propensity score, which included comorbidity, utilization, and diagnosis of CDI in the ICU as a proxy for disease severity. Patients were followed for recurrence within 8 weeks and for mortality at 30 days after infection.

## Results

There were 2,104 CDI patients treated with vancomycin matched with 8,014 CDI patients treated with metronidazole. Overall, 1,674 (16.5%) patients experienced a recurrence and 1,177 (11.6%) of patients died within 30 days. There was no difference in the risk of recurrence by treatment group (15.8% vs. 16.7% for vancomycin and metronidazole, p=0.32), but fewer patients who received vancomycin died (8.6% vs. 12.4%, RR 0.69, 95% CI 0.59 – 0.80).

## Conclusion

Recurrence rates were similar between treatment groups, but the risk of 30-day mortality was significantly reduced among patients who received vancomycin. The choice of treatment for CDI has become increasingly complex with the number of options available, and evidence regarding clinical cure, recurrence, mortality, and cost must all be taken into consideration. If validated, our observation that vancomycin results in a lower mortality rate may justify the additional costs of treatment relative to metronidazole.

## Disclosure of interest

None declared.

